# Common stressful life events and difficulties are associated with mental health symptoms and substance use in young adolescents

**DOI:** 10.1186/1471-244X-12-116

**Published:** 2012-08-17

**Authors:** Nancy CP Low, Erika Dugas, Erin O’Loughlin, Daniel Rodriguez, Gisele Contreras, Michael Chaiton, Jennifer O’Loughlin

**Affiliations:** 1 Department of Psychiatry, McGill University, 1033 Pine Avenue West, Montréal, QC, H3A 1A1, Canada; 2 Centre de Recherche du Centre Hospitalier de l'Université de Montréal, 3875 Saint Urbain, Montréal, QC, H2W 1V1, Canada; 3 Department of Psychiatry, University of Pennsylvania, 3535 Market Street, Suite 4100, Philadelphia, PA, 19104, USA; 4 Department of Social and Preventive Medicine, University of Montréal, 3875 Saint Urbain, Montréal, QC, H2W 1V1, Canada; 5 Centre for Addiction and Mental Health, University of Toronto, 33 Russell Street, Toronto, ON, M5S 2S1, Canada; 6 Institut national de santé publique du Québec, 190 Crémazie Blvd. East, Montréal, QC, H2P 1E2, Canada

**Keywords:** Adolescence, Stress, Mental health, Substances, Depression, Anxiety

## Abstract

**Background:**

Stressful life events are associated with mood disorders in adults in clinical settings. Less described in the literature is the association between common life stressors and a wide range of psychopathology in young adolescents. This study uses a large non-clinical sample of young adolescents to describe the associations among worry or stress about common life events/difficulties, mental health and substance use.

**Methods:**

Data on lifetime stress or worry about common life events/difficulties (i.e., romantic breakups, family disruption, interpersonal difficulties, and personal stress (health, weight, school work)), symptoms of depression, conduct disorder symptoms, and substance use were collected from 1025 grade 7 students (mean age 12.9 years; 45% male). The association between each source of stress and each mental health and substance use indicator was modeled in separate logistic regression analyses.

**Results:**

The proportion of adolescents reporting worry or stress ranged from 7% for new family to 53% for schoolwork. Romantic breakup stress was statistically significantly associated with all the mental health and substance use indicators except illicit drug use. Family disruption was statistically significantly associated with depression symptoms, marijuana use, and cigarette use. Interpersonal difficulties stress was statistically significantly associated with depression symptoms. All sources of personal stress were statistically significantly related to depression symptoms. In addition, health-related stress was inversely related to binge drinking.

**Conclusion:**

Young adolescents may benefit from learning positive coping skills to manage worry or stress about common stressors and in particular, worry or stress related to romantic breakups. Appropriate management of mental health symptoms and substance use related to common stressful life events and difficulties may help reduce emerging psychopathology.

## Background

Mental health and substance use disorders are among the most common health problems that affect adolescents, with prevalence estimates in population-based surveys as high as 15% for depression and 10% for substance misuse [1,2]. These disorders are associated with serious co-morbidity including underachievement in educational domains, poor socialization, delays in acquiring age-appropriate social skills, delinquency, and an elevated risk of suicide [3,4]. Mental health and substance use disorders often have their first manifestation during adolescence, yet frequently go undetected and untreated. Recurrence and chronicity of mental health disorders are high when onset occurs during adolescence [5].

The impact of stress on mental health and substance use disorders has been investigated for over 30 years [6,7]. Stressful life events are described as discrete quantifiable circumstances that can have severe negative impact. Severe traumatic events such as childhood physical abuse, assault, and rape, although less common, have been examined extensively in relation to post-traumatic stress disorder [8,9]. More prevalent stressful life events in adolescents include among others, parental divorce, a newly blended family, and changing schools or homes. Daily stressors do not necessarily relate to a specific event and include difficulties in relationships with boy/girlfriends, friends, family members, schoolwork, weight, and health problems such as asthma and acne. In addition to the impact on mental health, both stressful life events and daily stressors are associated with the onset and exacerbation of chronic illness (i.e., diabetes, asthma) [10-14], poor academic functioning, school absenteeism, high utilization of school services, and suicide [15].

Despite numerous cross-sectional and longitudinal studies, the relationship between stressful life events and mental health in adolescents is not yet fully understood [16-21]. In adults, stressful life events often precede mental health problems [22], whereas in adolescents, in addition to the same pattern, mental health symptoms (such as depressive symptoms) can lead to stressful life events (such as a romantic breakup) [23,24]. Specific gaps in the literature include: inconsistencies between studies that report no association between stressful events and depressive symptoms [20,25-27] and those that do [28-35], use of clinical samples which limit generalizability of the findings, examination of few and/or uncommon stressors (i.e., parental death, serious accident or illness), less emphasis on more prevalent, daily stressors [36], and a limited range of mental health and substance use outcomes. In addition, examination of specific age groups such as young adolescents, is still lacking. To address these gaps, the objective of this study was to describe the association between common daily and life stressors and indicators of mental health and substance use in a population-based sample of young adolescents.

## Methods

AdoQuest is a prospective cohort investigation of grade 5 students aged 10–12 years at cohort inception designed to investigate the natural course of the co-occurrence of health-compromising behaviours in children. The sample was drawn from a stratified sample of schools selected from among all French-language schools with more than 90 grade 5 students, located in the greater Montreal area. To assure equal representation of students across socioeconomic status (SES), all schools located in the target territory were stratified using a continuous SES indicator [37] and 9–10 schools were randomly selected from within each of the upper, middle and lower SES tertile groupings. Participants were recruited from all grade 5 classes in each of the 29 participating schools. Participants provided written assent and parents/guardians provided written informed consent. The study received ethics approval from the Faculty of Medicine of McGill University, the Conseil sur l’éthique et la recherche, Concordia University and the Centre de Recherche du Centre Hospitalier de l’Université de Montréal.

Data for this cross-sectional analysis were collected in 2006–7 when students were aged 11–15 years and in their first year of secondary school (grade 7). Data on socio-demographic characteristics, depression, stress, and substance use were collected in classroom-administered or mailed self-report questionnaires completed by 1025 of the 1631 original participants (63%). The low response relates to challenges following up students from 29 elementary schools as they transitioned to more than 100 secondary schools. Parents completed a self-report questionnaire (either brought home by participants or mailed to participants’ homes, and then returned by mail), which collected data on parents’ socio-demographic characteristics, lifestyle habits and health.

### Study variables

The measure of lifetime stress or worry related to specific life events and relationships and incorporated cognitive appraisal of stress, according to Lazarus and Folkman’s Transactional Model of Stress [38]. The measure reflects the tendency to worry or perceive an event as stressful. Data were collected in 12 items used previously in population-based surveys of Quebec adolescents [39]. Participants reported if they had ever in their lifetime been stressed or worried (not at all, a little bit, quite a bit, a whole lot; not applicable) by any of: (1) your parents separating or divorcing; (2) breaking up with your boyfriend or girlfriend; your relationship with your (3) father; (4) mother; (5) brother(s)/sister(s); (6) friends; (7) a health problem (such as acne or asthma); (8) your weight; (9) sexual relations; (10) your new family (i.e., a reconstituted family); (11) financial problems in your family; and (12) school work. For analyses, responses were dichotomized into no (not at all, not applicable) or yes (a little bit, quite a bit, a whole lot), as in previous studies using this measure [40]. It is well-established that divorce and blended families are associated with mental health symptoms and substance misuse in adolescence [41-46], and therefore the two items: “your parents separating or divorcing” and “your new family (i.e., a reconstituted family)” were combined into a single “family disruption” stress variable. Participants were categorized as having family disruption stress if their response was “yes” to either item. Due to accumulating evidence that stress related to romantic involvement during adolescence, and in particular during early adolescence, may also be associated with mental health symptoms, the item “breaking up with your boyfriend or girlfriend” was analyzed as a single item called “romantic breakup” stress [43,47-49]. A principal axis factor analysis with direct oblimin (with delta = 0.0) as the method of extraction, was conducted with the remaining 9 items. The Kaiser-Guttman eigenvalue criterion and scree tests suggested a 2-factor solution. Two items (i.e., sexual relations stress, financial problems stress) did not relate strongly to either factor in the pattern matrix, and were dropped from further analyses. Based on these results and conceptual reasoning, Factor 1 had 4 items (your relationship with your father; mother; brother(s)/sister(s); friends) and was labelled "interpersonal” stress. Factor 2 had 3 items (health problem; weight; school work) and was labelled "personal” stress. Since Cronbach’s α for the personal stress factor was 0.55, we analyzed each item within the factor separately.

Depression symptoms were measured in a validated 6-item scale [50] which assessed how often (never, rarely, sometimes, often) in the past 7 days participants: (1) felt too tired to do things; (2) had trouble going to sleep or staying asleep; (3) felt unhappy, sad, or depressed; (4) felt hopeless about the future; (5) felt nervous or tense; (6) worried too much about things. Responses were summed and then divided by the number of items responded to, to create a depression symptom score which ranged from 1 to 4 (mean (*sd*) = 1.8 (0.7); median = 1.7, α = 0.82), with higher values indicating more frequent symptoms. For multivariable analysis, the score was dichotomized at 1 standard deviation above the mean of the sex-specific distributions (2.7 for girls; 2.3 for boys) [51], which resulted in 15.2% of participants being labeled as having depression symptoms. The remaining 84.8% were labeled as not having depression symptoms.

Presence or absence of conduct disorder symptoms was assessed in a 15-item screening scale based on the DSM-IV criteria for diagnosing conduct disorder [52]. Participants reported any of the following (yes, no) in the past six months: (i) I have stayed out at night (until 4 or 5 am) despite my parents’ prohibitions; (ii) I have run away from home at least twice; (iii) I often bullied, threatened or intimidated others; (iv) I often initiated physical fights; (v) I have used a weapon (e.g., a knife, gun, chain, fist, bat, broken bottle) in a fight or to scare someone; (vi) I have been physically cruel to people; (vii) I have been physically cruel to animals; (viii) I have stolen while confronting a victim (e.g., mugging, purse snatching, extortion, armed robbery); (ix) I have forced someone into sexual activity; (x) I have deliberately engaged in fire setting with the intention of causing serious damage or to hurt someone; (xi) I have deliberately destroyed others’ property (objects, cars, buildings, broke windows); (xii) I have broken into someone else’s house, building, or car; (xiii) I often lied to obtain goods or favours or to avoid obligations; (xiv) I stole items of nontrivial value without confronting a victim (e.g., shoplifting; forgery); (xv) I was often truant from school. Participants were categorized as having conduct disorder symptoms if they responded yes to ≥3 of the 15 items. Cigarette smoking was assessed by asking participants if they had smoked a cigarette, even just a puff, in the past 6 months (yes, no). There is evidence of test-retest reliability of self-report smoking in youth [53], and there is strong agreement between self-reports and biochemical measures of tobacco use in prospective longitudinal studies, although recall beyond 1-year may be inconsistent [54,55].

Questions used to measure alcohol, marijuana and illicit substance use were drawn from two population-based surveys in Canada [56,57]. Binge drinking was assessed by asking participants to report the frequency of drinking ≥5 alcoholic drinks on the same occasion in the past 6 months [58,59]. Responses (I have never drunk alcohol; I have never drunk ≥5 drinks on one occasion; I have drunk ≥5 drinks on one occasion, but not in the 6 months; 1–2 times; 3–5 times; 6–9 times; 10–19 times; 20–39 times; ≥40 times) were dichotomized into yes (any report of consuming ≥five alcoholic drinks on one occasion in the past 6 months) or no. Binge drinking dichotomized in this manner has been associated with risky health behaviours in youth [60,61]. Test-retest reliability of self-report alcohol and other drug use measures, including marijuana, is high and use of short recall periods and simple language in stem questions leads to more accurate reporting [54].

Marijuana use was assessed by asking participants to report the frequency of consuming marijuana in the past 6 months. Responses (I have never consumed cannabis in my entire life; 1–2 times; 3–5 times; 6–9 times; 10–19 times; 20–39 times; ≥40 times; I don’t know what cannabis is) were dichotomized into yes (any marijuana use in the past 6 months) or no.

Use of illicit substances was measured by: “In your life, how often did you use the following drugs…(i) cocaine (coke, snow, crack, free base, powder); (ii) ecstasy (MDMA, E, X); (iii) hallucinogens (LSD, PCP, MESS, mushrooms, acid, mescaline, blotters); (iv) heroin (smack); (v) amphetamines (speed, upper); (vi) Ritalin (but NOT for medical reasons); (vii) tranquilizers taken without a prescription (downers, valium, Librium, dalmane, halcyon, ativan); (viii) steroids (testosterone, growth hormones, Dianobol, juice); (ix) Other drugs or medications taken without a prescription from a doctor?”. Response choices included: I have not used; just once to try; less than once a month (on occasion); about once a month; on the weekend OR 1–2 times a week; 3 or more times per week BUT not every day; every day. Because of the low endorsement of most items, we grouped all items into a single variable labelled “illicit drugs”. Participants were categorized as either yes (i.e., ever used one or more illicit drug) or no (i.e., never used any illicit drug).

Pubertal stage, assessed using the Pubertal Development Scale [62,63], was classified as: (i) “pre-pubertal” if in girls, there was no body hair growth, no menstruation and no breast growth, or in boys, there was no body hair growth, no facial hair growth, and no deepening of the voice; (ii) “pubertal” if, in girls, there was any indication of body hair growth, breast growth or menstruation, or in boys, if there was any indication of body hair and/or facial hair growth and/or voice changes; or (iii) “post-pubertal” if in girls, body hair growth and breast growth were completed and menstruation was reported; or in boys, body and facial hair growth, and voice changes were completed.

Socio-demographic data included age and sex (from the student questionnaire), and mother completed university (yes, no), and annual household income (<$30,000CAN, $30,000-$99,999, ≥$100,000, missing) (from the parent questionnaire).

### Analysis

The association between each stressor and each mental health (depression symptoms, conduct disorder symptoms) and substance use (binge drinking, cigarette smoking, marijuana use, and illicit drug use) indicator was investigated univariately. We used ANOVA for comparison of continuous variables, and chi-square tests for comparison of categorical variables. The association between each source of stress and each mental health and substance use indicator was modeled in separate logistic regression analyses. The models were adjusted for sex (i.e., the only potential confounder which was correlated consistently with the predictor variables of interest at r≥0.20), and each of the other stress indicators. In early models, we tested for possible interactions between sex and each stressor for each outcome. Only one of the 24 interaction terms tested was statistically significant (i.e., the sex X personal stress interaction term for smoking; p<0.0208). Because this finding could have occurred by chance, because we had no a priori hypotheses about this particular association and for consistency across analyses, we pooled the data across sexes for all analyses. All statistical analyses were performed using PAWS version 18.

## Results

The mean (*sd*) age of participants was 12.9 (0.4) years; 45% of the sample was male. Twenty-eight percent of participants had mothers who were university-educated. The families of 9% of participants had an annual household income less than $30,000; 63% had an annual household income between $30,000-$99,999, and 28% had an annual household income more than $100, 000. Ten percent (10.2%) of participants were pre-pubertal, 70.3% were pubertal, and 1.2% were post-pubertal (18.3% were missing data).

Participants retained for analysis were similar to those not retained in age, sex, and family income, but were more likely than those not retained to have mothers who were university-educated (Table1).

**Table 1:** Comparison of selected baseline characteristics between participants retained for analysis and those lost to follow-up. AdoQuest 2005.

	**Retained (n = 1025)**	**Lost to follow-up (n = 632)**	**p-value for difference**
Girls, %	55.4	53.2	0.414
Age, mean (*sd*)	10.8 (0.5)	10.8 (0.6)	0.090
Mother university-educated, %	27.9	18.4	0.000
Annual household income (CAN$)*, %			
<30,000	9.3	8.8	0.996
30,000-99,999	62.5	61.9	
≥100,000	28.2	29.3	

* Excludes participants with missing data.

The proportion of participants reporting that specific life events or difficulties had caused them stress or worry ranged from 7% for new family (i.e., a reconstituted family) to 53% for schoolwork (Table2). A statistically significantly higher proportion of girls than boys reported worry or stress for most stressors.

**Table 2:** Proportion of participants who reported ever in their lifetime being worried or stressed by specific life events or difficulties. AdoQuest, 2007.

	Ever worried or stressed*			
	**Total (n=1025) %**	**Girls (n=564) %**	**Boys (n=461) %**	**p-value for difference by sex**
Romantic breakup	21.3	22.7	19.5	0.221
Family disruption				
Divorce/separation	18.7	21.1	15.8	0.036
New family	7.1	9.0	4.8	0.010
Interpersonal stress				
Relationship with father	22.3	25.9	18.0	0.003
Relationship with mother	19.2	21.3	16.7	0.067
Relationship with siblings	26.3	30.0	21.9	0.004
Relationship with friends	34.3	40.8	26.5	0.000
Personal stress				
Health problem	25.8	30.9	19.5	0.000
Weight	34.8	42.0	26.0	0.000
School work	53.1	59.9	44.7	0.000

*Includes participants who reported that they were worried or stressed a little bit, quite a bit, a whole lot.

The prevalence of elevated depression and conduct disorder symptoms was 15% (by design) and 6%, respectively with statistically significant differences by sex for conduct disorder (Table3). Substance use did not differ by sex, and ranged from 3% (marijuana use in the past 6 months) to 9% (cigarette smoking in the past 6 months).

**Table 3:** Mental health symptoms and substance use according to sex, AdoQuest 2007

	**Total ( *n* =1025)**	**Girls ( *n* =564)**	**Boys ( *n* =461)**	**p-value for difference by sex**
**Mental health symptoms**				
Depression, %	15.2	14.4	16.3	0.432
Conduct disorder, %	5.7	4.5	7.4	0.050
Substance use				
Binge drank in past 6 months, %	4.4	4.6	4.1	0.761
Used marijuana in past 6 months, %	3.1	2.9	3.4	0.641
Cigarette smoking in past 6 months , %	8.9	9.6	8.0	0.440
Ever used illicit drugs, %	3.9	4.4	3.3	0.418

Univariately, all sources of stress were associated with depression and conduct disorder symptoms, and many were associated with substance use indicators (Table4). Multivariately, romantic breakup stress was statistically significantly associated with both depression and conduct disorder symptoms, and with all substance use indicators except illicit drugs (Table4). Family disruption stress was associated with depression symptoms, and both cigarette and marijuana use. The interpersonal stress factor was statistically significantly associated with depression symptoms. All three personal stress items (health, weight, schoolwork) were statistically significantly associated with depression symptoms and in addition, the health item was statistically significantly inversely associated with binge-drinking.

**Table 4:** Crude and adjusted odds ratios (OR) for the association between sources of worry or stress and indicators of mental health and substance use (n=1025). AdoQuest 2007.

	**OR**_**crude**_**(95% CI)**	**p-value**	**OR**_**adj**_(95% CI)*	**p-value**
**Depression**				
Romantic breakup	2.6 (1.8-3.8)	0.000	1.6 (1.1-2.4)	0.018
Family disruption	2.7 (1.8-3.9)	0.000	1.6 (1.0-2.4)	0.031
Interpersonal	4.8 (3.2-7.3)	0.000	2.7 (1.7-4.3)	0.000
Personal Stress				
Health	2.7 (1.9-3.9)	0.000	1.5 (1.0-2.3)	0.037
Weight	2.8 (2.0-3.9)	0.000	1.6 (1.1-2.4)	0.014
School work	3.9 (2.6-5.9)	0.000	2.2 (1.4-3.5)	0.001
**Conduct disorder**				
Romantic breakup	4.4 (2.6-7.5)	0.000	3.4 (1.9-6.0)	0.000
Family disruption	2.1 (1.2-3.7)	0.011	1.3 (0.7-2.4)	0.395
Interpersonal	2.7 (1.5-5.0)	0.001	1.7 (0.9-3.4)	0.123
Personal Stress				
Health	1.8 (1.0-3.1)	0.039	1.2 (0.6-2.1)	0.621
Weight	1.8 (1.1-3.1)	0.032	1.3 (0.7-2.3)	0.432
School work	2.2 (1.2-3.9)	0.009	1.4 (0.7-2.7)	0.291
**Binge drank in past 6 months**				
Romantic breakup	6.1 (3.3-11.4)	0.000	6.9 (3.5-13.3)	0.000
Family disruption	1.4 (0.7-2.8)	0.330	1.0 (0.5-2.1)	0.992
Interpersonal	1.5 (0.8-2.8)	0.175	1.2 (0.6-2.4)	0.684
Personal Stress				
Health	0.5 (0.2-1.2)	0.109	0.3 (0.1-0.7)	0.009
Weight	1.8 (0.2-1.2)	0.051	1.8 (0.9-3.5)	0.098
School work	0.9 (0.5-1.6)	0.727	0.6 (0.3-1.3)	0.189
**Use marijuana in past 6 months**				
Romantic breakup	4.2 (2.0-8.6)	0.000	3.7 (1.7-8.1)	0.001
Family disruption	3.2 (1.6-6.8)	0.001	2.7 (1.2-6.1)	0.017
Interpersonal	1.6 (0.8-3.4)	0.203	0.9 (0.4-2.3)	0.880
Personal Stress				
Health	1.0 (0.4-2.3)	0.997	0.7 (0.3-1.6)	0.335
Weight	2.3 (1.1-4.7)	0.023	2.1 (0.9-4.7)	0.073
School work	0.9 (0.5-1.9)	0.825	0.6 (0.3-1.3)	0.189
**Cigarette smoking in past 6 months**				
Romantic breakup	4.1 (2.6-6.3)	0.000	3.4 (2.1-5.4)	0.000
Family disruption	2.7 (1.7-4.2)	0.000	1.9 (1.2-3.2)	0.010
Interpersonal	2.0 (1.3-3.1)	0.003	1.1 (0.7-1.0)	0.649
Personal Stress				
Health	1.5 (0.9-2.3)	0.100	0.9 (0.6-1.6)	0.782
Weight	1.9(1.2-2.9)	0.005	1.3 (0.8-2.1)	0.289
School work	1.5(1.0-2.4)	0.053	1.0 (0.6-1.7)	0.966
**Ever used illicit drugs**				
Romantic breakup	2.2 (1.0-4.6)	0.045	1.5 (0.7-3.4)	0.290
Family disruption	2.5 (1.2-5.3)	0.014	1.8 (0.8-3.9)	0.156
Interpersonal	2.6 (1.2-5.7)	0.018	1.7 (0.7-4.3)	0.234
Personal Stress				
Health	2.1 (1.0-4.4)	0.040	1.4 (0.6-3.2)	0.367
Weight	1.4 (0.7-2.8)	0.395	0.8 (0.3-1.7)	0.513
School work	1.9 (0.9-4.0)	0.104	1.2 (0.5-2.8)	0.705

*ORs adjusted for sex and all other sources of stress.

**n ranged from 987 to 1022 due to missing data.

## Discussion

In this analysis, we examined the relationships between common sources of worry or stress, mental health symptoms and substance use in a population-based sample of young adolescents. In contrast to previous research that typically examined only 1–2 stressors in relation to a single outcome, our approach examined 6 sources of stress in relation to several mental health and substance use outcomes.

We found that adolescents reported worry or stress about a wide range of life events and difficulties. Two previous province-wide, school-based surveys of adolescents aged 12–18 years (n=1841 and n=2484, respectively) that used the same stress measure^19^, reported similar or lower proportions. Specifically, 22.5% and 20.3% (versus 21.3% in our study) reported romantic breakup stress; 5.7% and 5.8% (versus 18.7%) reported stress related to the divorce or separation of parents; 3.1% and 3.1% (versus 7.1%) reported stress related to a new family; 15.5% and 14.6% (versus 22.3%) reported stress related to relationship with father, 14.3% and 13.9% (versus 19.2%) reported stress related to relationship with mother; and 10.8% and 8.5% (versus 25.8%) reported stress related to a health problem. Our higher proportions may reflect that young adolescents find these life events and difficulties more stressful or worrisome than their older counterparts.

Consistent with previous reports [64,65], worry and stress were reported more frequently by girls than boys. This is likely related to well-established sex differences in how girls and boys cope with stress [66]. Girls tend to ruminate (i.e., passively and repetitively focus on their perceived failures) and use emotions to cope, both of which correlate highly with a “stressful reaction.” Boys are more problem-focused and use distraction to cope, both of which are unrelated to a “stressful reaction”.

It is notable that, with the exception of illicit drug use, romantic breakup stress was independently associated with all outcomes investigated. This finding is consistent with a growing literature suggesting that romantic involvement and breaking up in young adolescents is associated with depression, anxiety and conduct disorders [43,47,49,67,68]. Romantic relationships at age 12–13 may be a particularly salient source of stress because of the tendency among younger adolescents to over attribute (i.e., “blame”) current mental symptoms to romantic relationships, rather than other potential concomitant stressors, such as problems with friends or schoolwork. The high emotional salience of romantic relationships in the adolescent mindset may reflect a lack of romantic experience and maturity, which may be associated with “catastrophic” reactions to relatively minor upsets in romantic relationships.

Similarly, young adolescents may also be particularly sensitive to stress related to other personal relationships. The finding that interpersonal stress was associated with depression is consistent with recent literature suggesting a longitudinal bi-directional association between depressive symptoms and interpersonal relationship problems [69,70].

Family disruption stress was associated with depressive symptoms, which confirms previous reports and may reflect the effects of a detrimental familial environment where young adolescents spend the majority of their time [41-43,71-73]. Parental or family problems may decrease the family attachment felt by adolescents, which could lead to an increase in involvement with substance-using peers [74,75]. Cigarette and marijuana use have been associated with parental discord, separation, and divorce in the few extant studies in non-clinical adolescent populations [51,76-79]. It is also possible that adolescents use cigarettes and marijuana for their mood-lifting and anxiolytic effects [80,81] or as an avoidance or escape mechanism from family problems at home.

Not surprisingly, all three sources of personal stress (health, weight and schoolwork), which relate to physical or intellectual traits, were associated with depression symptoms. This supports the conceptual model of depression that posits self-blame or attribution of negative events (viewed as “personal failures”) as triggers of depression [82]. Weight [83-85], acne [86] and school work [87] (which adolescents may view as personal failures) have each been reported to be associated with anxiety and depression.

Stress or worry about health was inversely associated with binge-drinking, perhaps reflective that many young adolescents are aware of, and perhaps fear, the detrimental health effects of alcohol use.

It is interesting that several sources of stress relate to a variety of diverse mental health and substance use indicators. Possible mechanistic underpinnings may relate to demands which require adaptation, placed on young adolescents by these stressors. While most adults have developed positive coping styles to manage these demands (i.e., seeking advice, obtaining information, accepting social support, internal reflection), younger adolescents who experience frequent stress for the first time, may not have yet developed appropriate coping skills, and instead use immature or negative coping styles such as withdrawal, avoidance or distraction with substance use [6,88-92]. There is some evidence to suggest that age 15 represents a turning point when youth begin to use more effective coping styles in response to stress [91].

Study limitations include its cross-sectional design which limits causal inference. While data on the exposures and outcomes were collected at the same time, the reference time frames varied. Exposure to stress or worry was measured using a lifetime time frame. The outcomes were measured using a “past 7 days” time frame (depression symptoms), a “past 6 months” time frame (conduct disorder symptoms, binge drinking, cigarette smoking, marijuana use) or a “lifetime time frame” (illicit drug use). Different reference time frames may have obscured the estimates. Lifetime illicit drug use may have been under-reported. Our stress measure may have reflected a tendency to have a “stressful or worrisome reaction” or to perceive an event as worrisome or stressful rather than an actual experience of worry or stress. Lastly, given that the sample was one of convenience and the loss to follow-up, our findings may be prone to selection bias.

## Conclusions

### Implications

The results of this current study suggest an important role for mental health clinicians. Screening adolescents aged 12–13 for stressful life events and difficulties, perhaps through the use of event checklists, may be warranted. If inquiry is positive, further elaboration on the event and its meaning or significance may be elicited. Screening for depression, anxiety and substance use to establish a baseline level of symptoms and to monitor symptoms over time may also be helpful. Mitigating factors between events and symptoms include social support and coping, and therefore, assessment of the adolescent’s social support system and the way he/she is coping with respect to the specific event or difficulty is important. Positive coping skills teaching can include healthy distraction (e.g. physical activity), problem-solving and techniques of healthy emotional regulation. If the stressful events are related to interpersonal difficulties, teaching, practicing and modeling (i.e., role play) of interpersonal skills may be useful. Skills include how to identify specific interpersonal problems, how to communicate effectively and how to problem solve [93]. Follow-up over 2–3 months may be helpful and should problems persist, using cognitive-behavioral techniques, such as teaching cognitive restructuring (i.e., targeting dysfunctional attitudes, pessimistic attributional style, attention/memory for negative emotional information) may be warranted.

Overall, parents, teachers and counselors should be vigilant regarding potential stressors in adolescents’ lives, and in particular, stress related to romantic breakups. They should provide help if mental health symptoms and problematic substance use persist, especially in view of recent longitudinal reports suggesting that poor adjustment to stressful events and relationships during adolescence may forecast poorer mental health in adulthood [94].

## Competing interests

The authors declare no competing interests.

## Authors’ contributions

The contribution of authors is as follows: NL conceived of the study question, reviewed the literature, contributed to the design of the analysis and interpretation of data, and wrote sections of the article. ED, DR, EOL, GC, MC reviewed the literature, contributed to the design of the analysis and interpretation of data, and wrote sections of the article. EOL, ED, DR & GC conducted the data analysis and drafted the data analysis section in the article. JOL designed the study, obtained the funding, developed the survey instruments, supervised data collection, contributed to the design of the analysis and interpretation of data, coordinated drafting the article, and critically reviewed and edited sections of the article. All authors reviewed the article and approved the final version.

## Pre-publication history

The pre-publication history for this paper can be accessed here:

http://www.biomedcentral.com/1471-244X/12/116/prepub
